# Biportal-RATS vs. Uniportal-VATS for Lung Resections: A Propensity Score-Matched Analysis from Early Experience

**DOI:** 10.3390/jcm14248715

**Published:** 2025-12-09

**Authors:** Dania Nachira, Khrystyna Kuzmych, Maria Teresa Congedo, Alessia Oddone, Giuseppe Calabrese, Alessia Senatore, Giovanni Punzo, Maria Letizia Vita, Leonardo Petracca-Ciavarella, Stefano Margaritora, Elisa Meacci

**Affiliations:** 1 Department of General Thoracic Surgery, Fondazione Policlinico Universitario “A. Gemelli”, IRCCS, Università Cattolica del Sacro Cuore, 00168 Rome, Italy; danynac@libero.it (D.N.); mariateresa.congedo@policlinicogemelli.it (M.T.C.); alessia.oddone2000@gmail.com (A.O.); giuseppe93calabrese@virgilio.it (G.C.); alessia.senatore01@icatt.it (A.S.); marialetizia.vita@policlinicogemelli.it (M.L.V.); leonardo.petraccaciavarella@policlinicogemelli.it (L.P.-C.); stefano.margaritora@policlinicogemelli.it (S.M.); elisa.meacci@unicatt.it (E.M.); 2 Department of Anesthesiology and Intensive Care Medicine, Fondazione Policlinico Universitario “A. Gemelli”, IRCCS, Università Cattolica del Sacro Cuore, 00168 Rome, Italy; giovanni.punzo@policlinicogemelli.it

**Keywords:** biportal robotic-assisted thoracic surgery (Bi-RATS), minimally invasive thoracic surgery, quality of life (QoL), robotic-assisted robotic thoracic surgery (RATS), lymphadenectomy

## Abstract

**Background/Objectives**: Minimally invasive thoracic surgery has evolved rapidly, with uniportal video-assisted thoracoscopic surgery (U-VATS) and robotic-assisted thoracic surgery (RATS). Biportal-RATS (Bi-RATS) has emerged as a hybrid technique, combining robotics advantages with the reduced invasiveness of U-VATS. The aim of this study was to evaluate the safety, perioperative outcomes, lymphadenectomy, and postoperative quality of life (QoL) of Bi-RATS compared with U-VATS for lung resections. **Methods**: This single-center, observational cohort study included 130 consecutive patients undergoing anatomical lung resection between December 2021 and December 2024. Baseline and perioperative characteristics, including complications, chest drain duration, hospital stay, and lymph node yield, were analyzed. Health-related QoL was assessed preoperatively and 6 months postoperatively using the EQ-5D-5L questionnaire and EQ-VAS. Propensity score matching (PSM) at a 1:1 ratio was performed to minimize selection bias, obtaining 32 patients per group. **Results**: After PSM, the baseline characteristics were comparable between groups. Operative time was longer with Bi-RATS (221.3 ± 84.5 vs. 119.3 ± 53.4 min, *p* < 0.001). No significant differences were observed in postoperative complications, drain duration, or hospital stay. Bi-RATS seemed to be associated with a higher lymph node yield, particularly in segmentectomies. At 6 months, the overall EQ-VAS was comparable between techniques (78.9 U-VATS vs. 78.1 Bi-RATS; *p* = 0.832), while among the EQ-5D-5L dimensions, only mobility favored Bi-RATS (*p* = 0.045). **Conclusions**: Bi-RATS appears safe and effective, with perioperative outcomes and overall EQ-VAS comparable to those of U-VATS 6 months after surgery. These findings suggest that Bi-RATS may represent a valuable evolution of minimally invasive thoracic surgery.

## 1. Introduction

Minimally invasive thoracic surgery has progressively transformed the management of lung cancer, offering reduced postoperative morbidity and faster recovery compared with open thoracotomy, while maintaining oncological adequacy. Among the minimally invasive approaches, uniportal video-assisted thoracoscopic surgery (U-VATS) and robotic-assisted thoracic surgery (RATS) have become the most widely adopted worldwide [1,2,3,4].

U-VATS has gained broad acceptance over the last decade as an evolution of multiportal VATS. By providing direct anterior visualization of the pulmonary hilum through a single small incision, U-VATS replicates the ergonomics of thoracotomy while minimizing chest wall trauma. Several studies have confirmed its safety, feasibility, and effectiveness for both standard and complex pulmonary resections, including segmentectomy and sleeve procedures [5,6]. The benefits of U-VATS include reduced postoperative pain, shorter hospital stays, faster functional recovery, and satisfactory cosmetic outcomes compared with conventional approaches [7,8,9].

RATS, in contrast, represents one of the most technologically advanced forms of minimally invasive thoracic surgery. The robotic platform provides high-definition three-dimensional vision, tremor filtration, and articulated instruments with seven degrees of freedom, enabling precise dissection even in challenging anatomical locations and facilitating intracorporeal suturing [10,11,12]. However, its broader adoption has been tempered by limitations such as increased costs, longer operative times, and the absence of tactile feedback [13,14]. Furthermore, conventional RATS generally requires three or four access ports, which may increase postoperative discomfort as compared with U-VATS [15]. While some studies suggest that RATS may provide advantages in lymph node dissection, particularly in complex settings [16], evidence remains heterogenous.

In this context, biportal robotic-assisted thoracic surgery (Bi-RATS) has been introduced as a hybrid that combines the ergonomic and optical advantages of robotics with the reduced invasiveness of U-VATS. By limiting access to two ports, Bi-RATS may reduce intercostal trauma and postoperative pain while allowing precise and safe surgical approach. Initial feasibility reports, mainly from Asian centers, suggest that Bi-RATS is both safe and effective [17,18]. However, comparative evidence remains scarce, and, importantly, data of patients’ quality of life are lacking.

The present study aimed to compare Bi-RATS with U-VATS in terms of perioperative outcomes and postoperative quality of life, assessed using validated instruments. To our knowledge, this represents one of the first studies to systematically evaluate quality of life in patients undergoing Bi-RATS versus U-VATS, thereby providing novel insights into the balance between surgical radicality and patient-centered recovery.

## 2. Materials and Methods

This is a single-center, observational cohort study conducted between December 2021 and December 2024.

The study was conducted in accordance with the principles of the Declaration of Helsinki and approved by the Institutional Review Board of Università Cattolica del Sacro Cuore (Approval Code: ID3921; Approval Date: March 2021). All procedures performed in this study were in accordance with the ethical standards of the institutional and national research committees. Written informed consent was obtained from the patients prior to participation for the publication of this study.

Consecutive patients referred to our department and deemed eligible for lung resections were prospectively enrolled. Patients were assigned to the two groups (Bi-RATS or U-VATS) according to the surgical technique performed by the operating surgeon and the availability of an operating room equipped with a Da Vinci Xi Surgical platform (Intuitive Surgical, Sunnyvale, CA, USA).

The exclusion criteria were as follows:

- Age < 18 years.
- Previous pulmonary or chest wall surgery.
- Prior neoadjuvant treatments.

### 2.1. Preoperative Assessment

All patients underwent a standardized preoperative work-up including blood examinations, pulmonary function tests, cardiological evaluation, and chest computed tomography (CT) with contrast. Whole-body positron emission tomography (PET) was performed when clinically indicated. Patients were evaluated by a multidisciplinary tumor board before surgery. The choice of surgical approach was made according to surgeon expertise, intraoperative planning, and platform availability. Both techniques were part of the standard institutional practice, and no randomization was applied.

### 2.2. Surgical Techniques

Following the induction of general anesthesia, single-lung ventilation was achieved using a double-lumen endotracheal tube to optimize exposure of the surgical field. Patients were then positioned in the lateral decubitus, with the operative hemithorax elevated.

Bi-RATS procedures were performed using the da Vinci Xi surgical system (Intuitive Surgical, Sunnyvale, CA, USA). The approach is characterized by two incisions. A 3–4 cm utility incision in the 5th intercostal space along the mid-axillary line, fitted with a wound protector, accommodating the 30° robotic camera and a primary robotic instrument. An accessory port, usually placed in the 7th–8th intercostal space along the anterior line, allowed the introduction of a second robotic instrument or staplers. This configuration allowed all resections to be performed entirely with robotic instrumentation, including robotic staplers of different sizes [19].

U-VATS resections were carried out through a single 3–4 cm incision, usually in the 5th intercostal space, with adjustments to the 4th or 6th space when required based on lesion location and the patient’s anthropometric features. A wound protector was routinely inserted, and a 30° thoracoscope placed in the upper part of the incision. Long, curved thoracoscopic instruments, with proximal and distal articulation, specifically designed for the technique, and endoscopic staplers were introduced through the same access point [1,20].

Among anatomical resections, segmentectomy was mainly performed in cases of small (<2 cm) peripheral or sub-solid nodules, or in patients with a previous malignancy and a small lung nodule suspected for metastasis or primary lung cancer without preoperative diagnosis. Systematic hilar and mediastinal lymph node dissection was routinely performed in all anatomical resections in cases of solid or part-solid NSCLC, irrespective of the approach.

Following surgery, the patients were transferred to a monitored recovery unit, where pain control, respiratory function, and hemodynamic stability were supervised. Enhanced recovery after surgery (ERAS) protocols were routinely applied to facilitate postoperative recovery. These include early mobilization, physiotherapy, and pulmonary exercises, and the use of regional anesthesia techniques (e.g., nerve and fascial plane blocks) for pain management when required [21].

In both approaches, a single 28Fr chest tube was placed at the end of the procedure—through the caudal incision in Bi-RATS and through the surgical access in U-VATS. The chest drain was generally removed on postoperative day 2 or 3, provided that output was <250–300 mL in the previous 24 h, no air leak was present, and the postoperative X-ray showed complete lung re-inflation.

### 2.3. Assessment of Health-Related Quality of Life (EQ-5D-5L)

Health-related quality of life (HRQoL) was assessed using the validated EQ-5D-5L questionnaire developed by the EuroQol Group [22]. This instrument comprises a descriptive system covering five dimensions—mobility, self-care, usual activities (e.g., work, leisure activities, and family), pain/discomfort, and anxiety/depression—each rated on five levels of severity (no problems, slight problems, moderate problems, severe problems, and extreme problems). Additionally, a visual analog scale (EQ-VAS) ranging from 0 (“worst health imaginable”) to 100 (“best health imaginable”), on which patients rated their overall health status at the time of assessment, was introduced. From the descriptive system, a single index value can be calculated using country-specific value sets.

Patients completed the EQ-5D-5L in the preoperative period and again six months after surgery. Responses were analyzed both dimension-by-dimension and as summary index values. This approach enabled a patient-centered comparison of the postoperative impact of Bi-RATS versus U-VATS, supplementing the analysis of traditional clinical outcomes.

### 2.4. Outcomes

The primary outcome of this study was to evaluate the safety and surgical efficacy of Bi-RATS compared with U-VATS, an established minimally invasive approach. Surgical outcomes of interest included the incidence of intra- and postoperative complications, duration of chest drainage, and length of hospital stay.

The secondary outcome was to assess health-related quality of life using the validated EQ-5D-5L instrument.

### 2.5. Statistical Analysis

Continuous variables were expressed as mean ± standard deviation (SD) when normally distributed or as median and interquartile range (IQR) otherwise. Normality was assessed using the Shapiro–Wilk test. Comparisons between continuous variables were performed using Student’s *t*-test for independent samples (with adjustment for unequal variances when appropriate) or the Mann–Whitney U test for non-normal distributions.

Categorical variables were presented as absolute numbers and percentages and compared using the Chi-square test.

To account for the non-randomized nature of the study and balance the baseline characteristics of the two groups, propensity score matching (PSM) analysis was conducted with a 1:1 ratio using the nearest-neighbor matching method. Variables entered into the matching model included age, sex, body mass index (BMI), presence of comorbidities, and type of anatomical resection.

A repeated-measures ANOVA was conducted to assess changes over time, with Bonferroni correction applied for multiple comparisons.

A two-tailed *p*-value of <0.05 was considered statistically significant. Statistical analyses were performed using the IBM SPSS Statistics software, version 25.0 for Macintosh (IBM Corp., Armonk, NY, USA).

## 3. Results

A total of 130 patients were included in the analysis, 91 undergoing U-VATS and 39 undergoing Bi-RATS procedures. Baseline characteristics were generally comparable between the groups (Table 1). However, patients in the U-VATS group had a higher prevalence of comorbidities (72.5% vs. 48.7%, *p* = 0.009) and underwent wedge resections more frequently (64.8% vs. 30.8%, *p* = 0.001). Operative time was significantly longer with Bi-RATS compared to U-VATS (210.7 ± 79.9 vs. 98.4 ± 47.1 min, *p* < 0.001).

The perioperative outcomes are summarized in Table 2.

To account for baseline imbalances and potential bias related to non-randomization, particularly with respect to the presence of comorbidities and type of surgical resection performed, propensity score matching was performed with a 1:1 ratio. After matching, demographic and surgical characteristics were well balanced, yielding two matched cohorts of 32 patients each (Table 3).

After PSM, operative time remained significantly longer with Bi-RATS (221.3 ± 84.5 vs. 119.3 ± 53.4 min, *p* < 0.001). Intra- and postoperative complications, chest drain duration, and hospital stays were comparable between techniques (Table 4). A subgroup analysis was conducted on the small number of patients who underwent anatomical resections (only twenty per group) to evaluate the number of lymph nodes retrieved. In particular, in segmentectomies (seven cases per group), the total number of lymph nodes retrieval was significantly higher with Bi-RATS compared to U-VATS (8.86 ± 7.11 vs. 1.43 ± 1.62, *p* = 0.019), including both N1 (2.14 ± 1.574 vs. 0.57 ± 0.79, *p* = 0.036) and N2 (6.71 ± 5.99 vs. 0.86 ± 0.90, *p* = 0.025) station nodes (Table 4). Nodal upstaging was null in both groups. It must be stated that, while all patients who underwent Bi-RATS segmentectomy had a solid or part-solid NSCLC, two out of the seven patients in the U-VATS group had a lung metastasis and one had a pure ground-glass opacity (GGO); therefore, a systematic lymphadenectomy was not performed.

**Figure 1:**
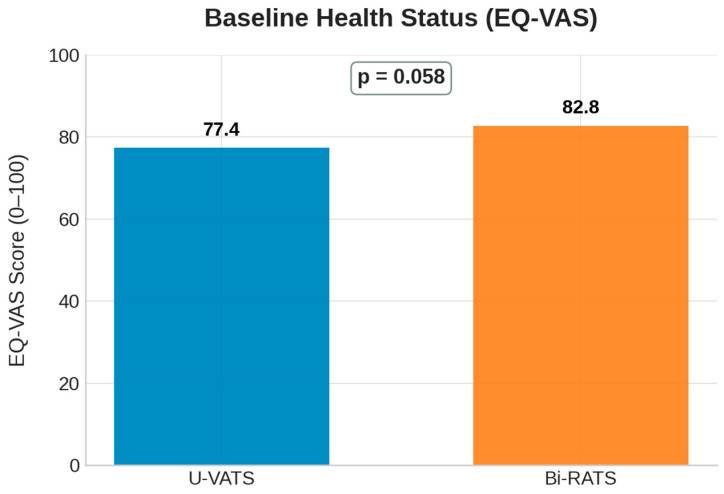
Baseline health status assessed by the EQ-VAS (0–100 scale) in patients undergoing U-VATS and Bi-RATS.

**Figure 2:**
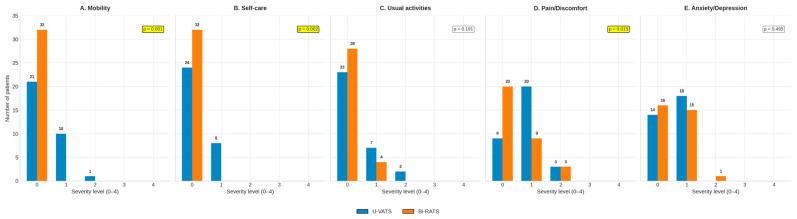
Baseline EQ-5D-5L dimensions in patients undergoing U-VATS and Bi-RATS. Distribution of responses across severity levels (0 = no problems; 4 = extreme problems) for (**A**) mobility, (**B**) self-care, (**C**) usual activities, (**D**) pain/discomfort, and (**E**) anxiety/depression. Significant differences between groups are indicated by *p*-values within the panels.

Preoperative EQ-5D-5L and EQ-VAS scores were comparable between groups (mean EQ-VAS *p* = 0.058; Figure 1). Based on the data provided by the patients before the surgery, it was also possible to analyze and compare the five parameters of the EQ-5D-5L between the two types of surgical approaches (Figure 2A–E):

1. Mobility.
2. Self-care.
3. Usual activities (work, study, and family).
4. Pain or discomfort.
5. Anxiety or depression.

**Figure 3:**
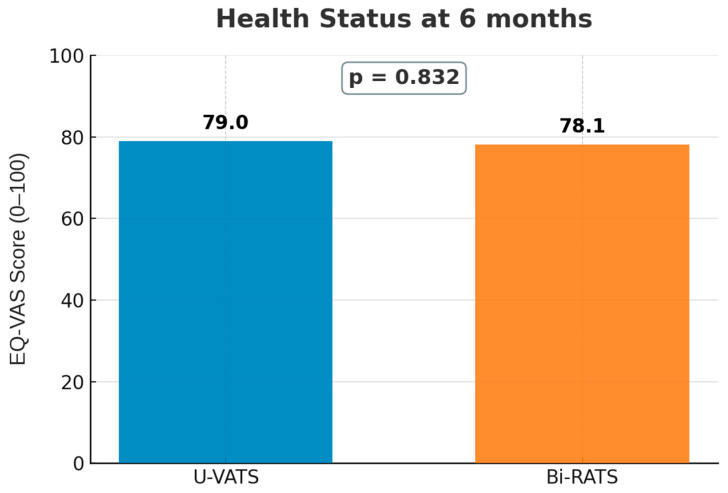
Health status 6 months after surgery. No significant difference was observed between the groups at 6 months (*p* = 0.832).

Six months after the surgery, the overall EQ-VAS values were similar between the groups (78.9 U-VATS vs. 78.1 Bi-RATS, *p* = 0.832; Figure 3).

**Figure 4:**
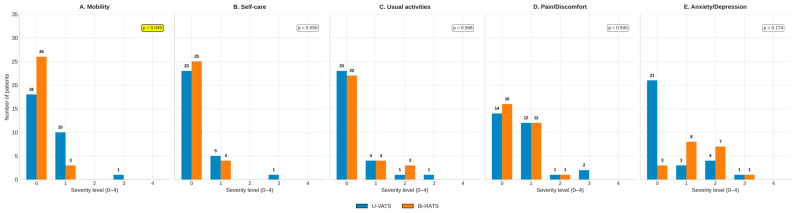
(**A**–**E**): EQ-5D domains 6 months after surgery between the two groups.

Domain-specific analysis at 6 months revealed the following patterns between techniques (Figure 4A–E). Bi-RATS showed better scores only in mobility (*p* = 0.045).

However, distinct patterns emerged when comparing the EQ-5D-5L domains between the baseline and at 6 months within each surgical technique.

**Figure 5:**
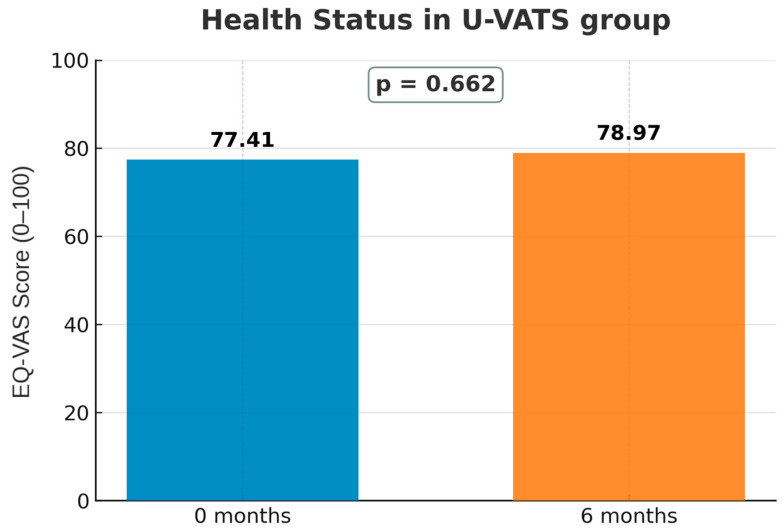
EQ-VAS score at baseline (0 months) and 6 months after U-VATS procedures.

**Figure 6:**
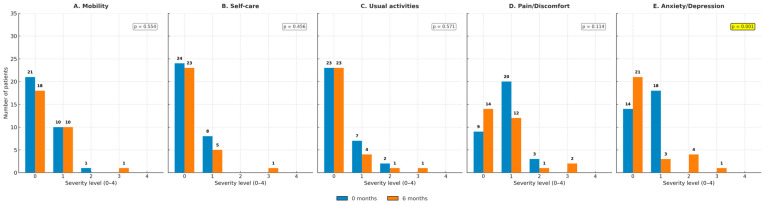
EQ-5D domains in the U-VATS group at baseline and 6 months for each dimension. (**A**) Mobility; (**B**) self-care; (**C**) usual activities; (**D**) pain/discomfort; and (**E**) anxiety/depression. In this cohort, a significant improvement was observed in anxiety/depression (*p* = 0.001), while no significant differences were detected in other domains.

For the U-VATS group, overall health perception (EQ-VAS) remained stable from baseline to 6 months (*p* = 0.662; Figure 5). Domain-specific analyses of EQ-5D-5L showed no significant changes in mobility, self-care, usual activities, or pain/discomfort (Figure 6A–D). In contrast, anxiety/depression significantly improved at 6 months (*p* = 0.001; Figure 6E).

**Figure 7:**
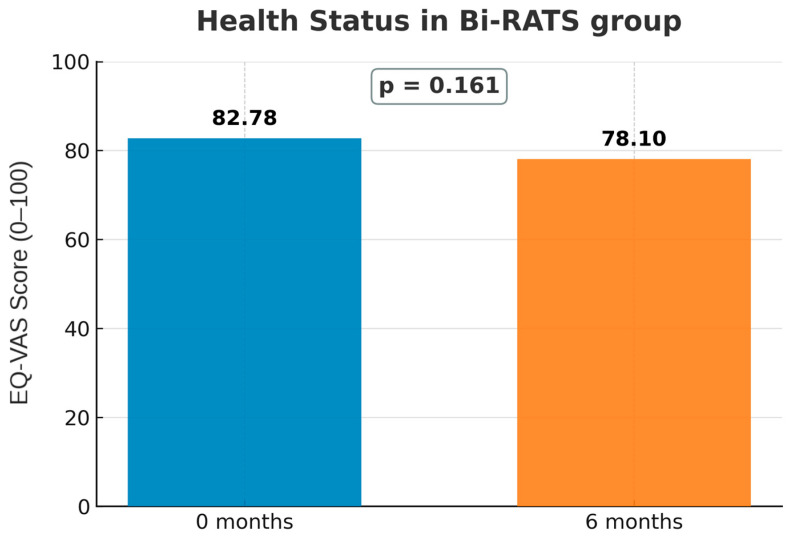
EQ-VAS score at baseline (0 months) and 6 months after Bi-RATS procedures.

**Figure 8:**
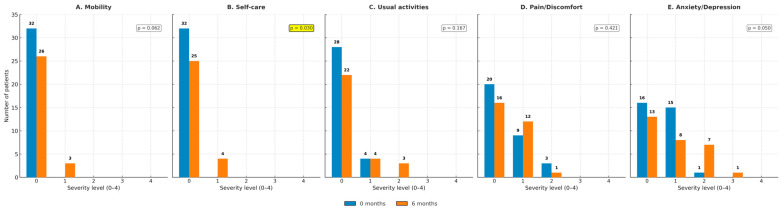
EQ-5D domains in the Bi-RATS group at baseline and 6 months for each dimension. (**A**) Mobility; (**B**) self-care; (**C**) usual activities; (**D**) pain/discomfort; and (**E**) anxiety/depression. In this group, significant differences were observed for self-care (*p* = 0.030) and anxiety/depression (*p* = 0.050), while other domains did not show a significant change over time.

In the Bi-RATS group, overall health perception showed a non-significant decline from baseline to 6 months (*p* = 0.161; Figure 7). Domain-level EQ-5D-5L analyses indicated worsening in self-care (*p* = 0.030) and a slight deterioration in mobility (*p* = 0.062). Anxiety/depression also trended towards deterioration (*p* = 0.050), while usual activities and pain/discomfort remained stable (Figure 8A–E).

## 4. Discussion

Minimally invasive thoracic surgery has evolved significantly over the last two decades, with uniportal-VATS and robotic-assisted techniques representing two of the most advanced approaches currently available. While U-VATS is widely recognized for its reduced invasiveness, the robotic platform offers unique advantages in terms of instrument articulation, tremor filtration, and three-dimensional visualization [10,23]. In this context, our study aimed to compare biportal-RATS (Bi-RATS) with U-VATS in terms of safety, perioperative outcomes, lymphadenectomy, and patient-reported quality of life.

Our results show that Bi-RATS seems to be a safe and effective technique, with postoperative complication rates, drain duration, and hospital stay comparable to U-VATS. These findings are consistent with the previous literature reporting no excess morbidity associated with robotic approaches [24,25]. Operative times were longer in the Bi-RATS group, in line with prior studies, and this is largely attributed to docking, robotic arm alignment, and instrument exchange. It should be noted that all of these steps can be significantly optimized over time as the center gains experience with a new technique. Our data, in fact, should be interpreted with caution, taking into account that they encompass the learning-curve period of Bi-RATS at our center.

Importantly, the disadvantages of the RATS technique appear balanced by the ergonomic and visual benefits of the robotic system, which may facilitate more precise dissection and reduce tissue trauma [26,27].

In this context, previous reports have suggested that robotic surgery may enhance lymph node harvest compared with VATS [28,29,30], particularly in technically challenging anatomical scenarios. Additionally, the completeness of nodal dissection is known to be a recognized determinant of staging accuracy and oncological radicality in non-small-cell lung cancer (NSCLC). Our limited data on this topic showed an interesting finding. Although no statistically significant differences emerged in lobectomies, there was a trend in favor of BI-RATS, with a higher total nodal yield (15.0 ± 10.8 vs. 8.5 ± 5.3; *p* = 0.062). The most significant differences, however, were observed in segmentectomies, where anatomical complexity makes lymphadenectomy particularly challenging, especially at the N1 stations. In these cases, Bi-RATS enabled a significantly larger excision on a limited series of seven patients: the total number of lymph nodes was significantly higher (*p* = 0.019), as well as the number of N1 lymph nodes (*p* = 0.036) and N2 lymph nodes (*p* = 0.025). However, it must be taken into account that the results could be invalidated by the three cases in the VATS group (two metastases and one pure GGO) in which a systematic lymphadenectomy was not performed, but only a sampling. It is also important to clarify that the higher number of retrieved nodes did not correspond to any nodal upstaging, which was absent in both groups, and would not, therefore, have influenced the potential administration of adjuvant therapies.

These findings suggest that the superior dexterity and visualization provided by the robotic platform may translate into more complete lymphadenectomy when dissection is technically demanding. Although promising, these results should be interpreted cautiously given the retrospective design and very limited sample size of the subgroup analysis.

With respect to postoperative quality of life, our findings indicate no significant differences between Bi-RATS and U-VATS groups in terms of overall EQ-VAS at 6 months. However, the comparison of EQ-5D-5L scores at 6 months with the baseline within each surgical approach revealed a different pattern: the Bi-RATS group showed a decline in self-care and a trend toward worsening mobility and anxiety/depression from baseline to 6 months, while the U-VATS group showed stability or improvement (notably in anxiety/depression). Although many unmeasured confounders can explain these results, a possible explanation can lay in the different expectations of patients undergoing the two techniques.

Indeed, it is common among surgeons to observe that patients scheduled for robotic surgery perceive the procedure as less invasive and, unconsciously, expect a quicker recovery. Therefore, the reality of outcomes and recovery period compared with the expectations can lead to a depressive state with a consequent reduction in self-care.

Both approaches yielded quite comparable outcomes in terms of EQ-5D-5L values for pain/discomfort at 6 months, showing that the Bi-RATS technique does not increase postoperative discomfort. This observation is consistent with recent reports. In our center, Bi-RATS is performed using the same access as U-VTS combined with an accessory incision, strategically placed along the mid- and anterior axillary line, where the intercostal spaces are wider. This configuration may contribute to lower pain perception, as patients experience less intercostal compression and traction than in traditional RATS [30,31]. Furthermore, the Bi-RATS approach has been associated in the literature with reduced pain compared with multiportal RATS [15,20,31], likely owing to the reduced number of intercostal accesses. Although our study did not directly compare these techniques, above all, in terms of pain scores at different time points, this aspect further reinforces the potential benefits of the biportal approach that must be further investigated.

Another strength of this work is the inclusion of validated patient-reported outcome measures (EQ-VAS and EQ-5D-5L). This dual focus on both perioperative outcomes and quality of life provides a more comprehensive evaluation of surgical impact.

Taken together, these results suggest that Bi-RATS combines the advantages of robotics with the low invasiveness of uniportal access, offering a technically advanced alternative without compromising safety or patient-reported outcomes. From a clinical perspective, the potential for improved lymphadenectomy in segmentectomy cases may have relevant implications for accurate staging and oncological radicality.

In addition, while prior studies have compared postoperative quality of life between RATS and VATS more broadly [20,28,30,32,33], to our knowledge, this is among the first studies to specifically examine QoL outcomes in patients undergoing Bi-RATS compared directly to U-VATS.

This study has several limitations. The single-center, non-randomized design, combined with the relatively small cohort size, limits its generalizability. In particular, while the PMS reduced selection biases, it also resulted in small size groups and, consequently, an underpowered analysis (1 − ß = 37%, according to the post hoc analysis). Indeed, the subgroup analysis of segmentectomy cases is based on very few patients, which prevents any generalization of the conclusions. Furthermore, the study period encompasses the learning curve for Bi-RATS techniques at our institution; therefore, the results presented are only preliminary and must be interpreted with caution.

Long-term oncological outcomes were not evaluated, and cost-effectiveness analyses were beyond the scope of this work.

Future research should include prospective, multi-center studies to validate the observed advantages of Bi-RATS in lymphadenectomy and to assess long-term survival and recurrence.

## 5. Conclusions

Within the limits of a preliminary study on a limited cohort, the biportal robotic approach seems to be a safe and effective technique, with surgical outcomes comparable to those of U-VATS. Notably, Bi-RATS seemed to have superior performance in complex settings such as segmentectomy, where the articulation and precision of the robotic platform enable more complete lymph node dissection, although the results need to be confirmed on a larger series. At the same time, overall patient-reported quality of life (in terms of EQ-VAS) at six months was comparable between groups. These findings suggest that Bi-RATS may represent an evolution of minimally invasive thoracic surgery, combining the oncological thoroughness of robotics with the reduced morbidity typical of uniportal access.

## Figures and Tables

**Table 1:** Baseline characteristics of patients undergoing U-VATS and Bi-RATS procedures.

Variable	U-VATS (n = 91)	Bi-RATS (n = 39)	*p*-Value
Sex (male)	42 (46.15%)	13 (33.33%)	0.175
Age (years)	67.19 ± 10.60	69.10 ± 12.88	0.378
BMI (kg/m^2^)	25.78 ± 4.79	25.40 ± 3.84	0.665
Current smoker	15 (16.48%)	9 (23.08%)	0.691
Pack-years	26.68 ± 30.38	22.03 ± 26.63	0.413
Hypertension	49 (53.85%)	22 (56.41%)	0.837
Coronary artery disease (CAD)	8 (8.79%)	7 (17.95%)	0.14
Comorbidities (≥1)	66 (72.53%)	19 (48.72%)	**0.009**
Other neoplasms	43 (47.25%)	15 (38.46%)	0.355
Type of resection:			**0.001**
● Wedge	59 (64.83%)	12 (30.77%)	
● Segmentectomy	15 (16.48%)	11 (28.21%)	
● Lobectomy	17 (18.68%)	16 (41.03%)	
Side of access (right)	51 (56.04%)	24 (61.54%)	0.561
CT lesion diameter (mm)	16.61 ± 9.69	20.18 ± 9.10	0.057
Histology:			0.085
● NSCL	38 (41.75%)	25 (64.10%)	
● Lung metastasis	41 (45.05%)	14 (35.90%)	
● Others	12 (13.20%)	0	
Histological lesion diameter (cm)	2.13 ± 1.26	2.33 ± 1.16	0.794

Notes: Values are presented as the mean ± standard deviation (SD) or as a number (percentage). BMI = body mass index; CAD = coronary artery disease; CT = computed tomography; and NSCLC = non-small-cell lung cancer. *CT lesion diameter* refers to the largest lesion dimension measured on a preoperative CT scan, whereas *histological lesion diameter* refers to the maximal dimension of the resected specimen on final pathology.

**Table 2:** Surgical outcomes of patients undergoing U-VATS and Bi-RATS procedures.

Variable	U-VATS (n = 91)	BI-RATS (n = 39)	*p*-Value
Intraoperative complications	/	/	/
Adhesions	7 (7.69%)	2 (5.13%)	0.598
Conversion	1 (1.10%)	0 (0%)	0.511
Postoperative complications	16 (17.58%)	6 (15.38%)	0.759
Drain duration (days)	3.92 ± 3.16	3.97 ± 2.81	0.930
Hospital stay (days)	3.70 ± 2.30	3.33 ± 1.18	0.343
Total LN stations	1.12 ± 1.46	2.36 ± 1.98	**<0.001**
LYMPH NODES			
● LOBES			
Total LN stations	3.06 ± 1.14	3.94 ± 1.29	**0.047**
Total number of LNs	8.12 ± 5.23	15.56 ± 12.01	**0.026**
N1 lymph nodes	3.82 ± 2.58	4.56 ±3.08	0.459
N2 lymph nodes	4.29 ± 3.61	9.00 ± 9.27	0.061
● SEGMENTS			
Total LN stations	1.73 ± 1.16	2.45 ± 1.51	0.181
Total number of LNs	4.20 ± 3.69	8.00 ± 6.18	0.066
N1 lymph nodes	1.20 ± 1.52	2.70 ± 1.83	**0.036**
N2 lymph nodes	2.27 ± 2.25	5.10 ± 5.57	0.088

Notes: Values are presented as the mean ± standard deviation (SD) or as a number (percentage). LN = lymph node. Dashes (–) indicate that no events were observed. Significant *p*-values (<0.05) are shown in bold.

**Table 3:** Baseline characteristics of matched patients in the U-VATS and Bi-RATS groups.

Variable	U-VATS (n = 32)	BI-RATS (n = 32)	*p*-Value
Sex (male)	12 (37.50%)	12 (37.50%)	1.000
Age (years)	64.47 ± 10.38	68.56 ± 12.26	0.280
BMI (kg/m^2^)	25.83 ± 6.07	25.20 ± 4.01	0.622
Current smoker	4 (12.50%)	9 (28.13%)	0.227
Pack/years	16.64 ± 22.40	24.59 ±28.03	0.234
Hypertension	15 (46.88%)	17 (53.13%)	0.617
Coronary artery disease (CAD)	4 (12.50%)	5 (15.63%)	0.719
Comorbidities (≥1)	19 (59.38%)	19 (59.38%)	1.000
Other neoplasms	12 (37.50%)	11 (34.38%)	0.794
Type of resection:			1.000
● Wedge	12 (37.50%)	12 (37.50%)	
● Segmentectomy	7 (21.88%)	7 (21.88%)	
● Lobectomy	13 (40.63%)	13 (40.63%)	
Side of access (right)	17 (53.13%)	20 (62.50%)	0.448
CT lesion diameter (mm)	17.07 ± 9.62	19.59 ± 8.53	0.282
Histology:			0.238
● NSCLC	25 (78.13%)	24 (75.00%)	
● Lung metastasis	7 (21.87%)	8 (25.00%)	
Histological lesion diameter (cm)	1.71 ± 1.11	2.33 ± 1.16	0.448

Notes: Values are presented as the mean ± standard deviation (SD) or as a number (percentage). BMI = body mass index; CAD = coronary artery disease; CT = computed tomography, and NSCLC = non-small-cell lung cancer. *CT lesion diameter* refers to the maximum lesion size measured on a preoperative CT scan, whereas *histological lesion diameter* refers to the largest dimension on pathology.

**Table 4:** Surgical outcomes of matched patients in U-VATS and Bi-RATS procedures.

Variable	U-VATS (n = 32)	BI-RATS (n = 32)	*p*-Value
Intraoperative complications	/	/	/
Adhesions	4 (12.50%)	2 (6.25%)	0.391
Conversion	/	/	/
Postoperative complications	6 (18.75%)	6 (18.75%)	1.000
Drain duration (days)	3.75 ± 1.93	4.19 ± 3.07	0.498
Hospital stay (days)	3.97 ± 2.01	3.41 ± 1.29	0.187
LYMPH NODES:			
● LOBES			
Total LN stations	3.31 ± 0.947	3.92 ± 1.19	0.157
Total number of LNs	8.46 ± 5.27	15.00 ± 10.83	0.062
N1 lymph nodes	4.38 ± 2.63	4.38 ± 3.18	1.000
N2 lymph nodes	4.08 ± 3.30	8.15 ± 7.53	0.086
Nodal upstaging	/	/	/
● SEGMENTS			
Total LN stations	1.14 ± 1.22	2.43 ± 1.40	0.091
Total number of LNs	1.43 ± 1.62	8.86 ± 7.11	**0.019**
N1 lymph nodes	0.57 ± 0.79	2.14 ± 1.574	**0.036**
N2 lymph nodes	0.86 ± 0.90	6.71 ± 5.99	**0.025**
Nodal upstaging	/	/	/

Notes: Values are presented as the mean ± standard deviation (SD) or as a number (percentage). LN = lymph node. Dashes (–) indicate that no events were observed. Significant *p*-values (<0.05) are shown in bold.

## Data Availability

The datasets generated and/or analyzed during the current study are available from the corresponding author on reasonable request.
